# Social mobility and parenting: Testing associations in a prospective longitudinal cohort study

**DOI:** 10.1093/chidev/aacaf050

**Published:** 2026-02-20

**Authors:** Samiha Islam, Sara R Jaffee, Jay Belsky, Robert J Hancox, Richie Poulton, Sandhya Ramrakha, Jasmin Wertz

**Affiliations:** Department of Psychology, University of Pennsylvania, Philadelphia, PA, United States; Department of Psychology, University of Pennsylvania, Philadelphia, PA, United States; Department of Human Ecology, University of California, Davis, CA, United States; Department of Preventive and Social Medicine, University of Otago, Dunedin, Otago, New Zealand; Dunedin Multidisciplinary Health and Development Research Unit, Department of Psychology, University of Otago, Dunedin, Otago, New Zealand; Dunedin Multidisciplinary Health and Development Research Unit, Department of Psychology, University of Otago, Dunedin, Otago, New Zealand; Department of Psychology, University of Edinburgh, Edinburgh, Scotland, United Kingdom

**Keywords:** parenting, intergenerational, cognitive stimulation, sensitivity, socioeconomic status

## Abstract

This study tested whether parents’ social mobility is associated with parent–child interactions. Data came from 719 Dunedin Parenting Study members (mean age: 32.7; 52.3% female, 90.2% New Zealand European ethnicity) who have been followed from birth to midlife and participated in parenting assessments with their 3-year-old children (50% female). Upwardly mobile parents provided more sensitive parenting and cognitively stimulating environments than parents from stable-low socioeconomic backgrounds, but less sensitive parenting and cognitively stimulating environments than parents from stable-high socioeconomic backgrounds. These results were not fully explained by pre-existing differences between parents in experienced parenting and childhood characteristics. Our findings underscore the importance of supporting families with fewer socioeconomic resources through a life-course and intergenerational approach to caregiving environments.

Parenting behaviors tend to be socioeconomically stratified, such that parents with higher socioeconomic status (SES) are more likely to adopt parenting behaviors linked to higher academic achievement and stronger language skills in children (Madigan et al., 2019; Pinquart, 2016). There are many reasons for SES-related differences in parenting behaviors, some having to do with the constraints on time and resources that some parents experience (Doepke et al., 2019; Yeung et al., 2002), some reflecting parents’ values and aspirations, and some reflecting parents’ efforts to prepare their children for the environments they currently face and the environments they think their children will face in the future (Lareau, 2002). Whilst parents may have good reasons for adopting specific parenting practices, there have been concerns that SES-related differences in parenting behaviors may, at the same time, contribute to the intergenerational transmission of social inequalities (Kalil & Ryan, 2024). To reduce inequities, efforts have been made to support parents from lower-SES backgrounds to better promote children's cognitive and social development.

Efforts to support parents have taken at least two forms. The first is to directly target parenting behaviors, for example, through interventions that train parents to engage in interactive reading practices with their children (Dowdall et al., 2020; Fikrat-Wevers et al., 2021) or use particular discipline strategies (e.g., reinforcing good behavior and reducing harsh discipline) (Leijten et al., 2017). A wealth of research has studied these interventions and documented their effects on parenting, including for parents in lower SES environments (Fikrat-Wevers et al., 2021). However, these efforts have also attracted criticism for expecting parents to change their parenting practices in the absence of any meaningful change in their socioeconomic circumstances. The second approach is more indirect (and, to some extent, addresses the critique of the first approach), targeting a family's economic circumstances through interventions such as cash transfers. The expectation underlying this approach is that decreases in financial hardship will facilitate changes in parenting. Studies evaluating these interventions suggest that increases in parental income support parents to purchase child-specific goods and invest in early learning activities in the first three years of life, as well as engage in more supervision of and enjoyable interactions with adolescents (Akee et al., 2018; Gennetian et al., 2024). However, previous work has mostly focused on parental income, leaving it less clear how changes in other aspects of parental SES may affect parenting behaviors. Parental occupational status in particular is important to examine because it tends to be a more stable proxy for access to economic resources compared to income, whilst also capturing broader aspects of social status, prestige, and other advantages (such a job security and autonomy) that are relevant to parenting (Bradley & Corwyn, 2002).

Here we sought to address this question by examining parenting among individuals who were socially mobile, i.e., who experienced a change in socioeconomic circumstances—as indexed by occupational status—from childhood to adulthood. Comparisons between individuals who are socially mobile versus those whose SES remains the same from childhood to adulthood can be informative about the potential impact of changes in SES, particularly when controlling for pre-existing differences between individuals that may influence social mobility. For example, previous research shows that individuals who moved from a lower SES in childhood to a higher SES in adulthood (i.e., upwardly mobile) have better mental and physical health compared to those who remain in a low SES from childhood to adulthood (Gugushvili et al., 2021; Islam & Jaffee, 2024). This finding remained even after accounting for pre-existing differences between these groups in mental and physical health, which may make it more likely for some people to achieve upward mobility in the first place and therefore pose a threat to causal inference. Mirroring this pattern, individuals who moved from a higher SES in childhood to a lower SES in adulthood (i.e., downwardly mobile) tend to have lower mental and physical health compared to those who remain in a higher SES throughout (Islam & Jaffee, 2024). Here, we used this same analytic approach to test whether social mobility in occupational status is also associated with individuals’ parenting. There is very little research on social mobility in relation to parenting, even though parenting is a potential pathway through which benefits of upward mobility may be transmitted across multiple generations and thereby break intergenerational cycles of disadvantage.

In generating predictions about how parental social mobility might be associated with parenting, we considered both an “economic resources” and a “sociocultural” pathway. Economic resources are emphasized in theoretical frameworks such as the Family Stress Model and the Family Investment Model (Conger et al., 1992; Conger & Donnellan, 2007). The Family Stress Model posits that in families exposed to economic hardship, parents are more likely to experience psychological distress, which may lead them to engage in harsher parenting (Conger et al., 1992). The Family Investment Model posits that families with greater economic resources can invest more financial, social, and human capital in their children (Conger & Donnellan, 2007). Although both models emphasize the role of income, we draw on them here to generate hypotheses about how changes in occupational status may affect parenting, because higher occupational status is typically associated with greater access to economic resources (Bradley & Corwyn, 2002). In addition to economic resources, sociocultural factors may also play a role. Upward mobility puts parents into contact with new social environments via workplaces, communities, or peer networks, in which norms around parenting may differ from those in their SES of origin. For example, Lareau (2002) documents how approaches to parenting differ between working-class and middle-class parents, with the former's parenting characterized by more unstructured play, greater use of directives and less negotiation with children, and the latter's by highly-structured scheduling of children's lives and more reasoning-based interactions. Based on the economic resources and sociocultural pathways, we predicted that upwardly mobile parents would provide more sensitive parenting and cognitively stimulating environments compared to parents who remained in a lower SES throughout childhood and adulthood. Mirroring this prediction, we hypothesized that downwardly mobile parents would provide less sensitive parenting and cognitively stimulating environments compared to parents who remained in a higher SES into adulthood.

The theory and research reviewed thus far focus on the role of parents’ current SES in parenting. Parenting, however, is not only shaped by current socioeconomic circumstances. Life-course perspectives on parenting, such as the determinants of parenting process model (Belsky, 1984; Belsky & Jaffee, 2006), predict that parents’ experiences during their childhood also affect their parenting. Consistent with these perspectives, research shows that parents’ own experienced parenting in their family of origin, as well as their childhood socioeconomic circumstances, shape their parenting behaviors (e.g., Belsky et al., 2005; Madden et al., 2015; McAnally et al., 2021). These findings suggest that over and above parents’ current SES, the experience of having grown up in lower-SES circumstances may have a lingering impact on the parenting of upwardly mobile individuals. A residual effect of childhood circumstances would also be consistent with findings in research on social mobility and health, which show that upwardly mobile individuals do not enjoy the same health benefits as those who had always been in a high SES (Chen et al., 2022; Islam & Jaffee, 2024; Simandan, 2018). Based on the life-course perspective, we predicted that upwardly mobile parents would provide less sensitive parenting and cognitively stimulating environments compared to parents who had always been in a high SES, and that downwardly mobile parents would provide more sensitive parenting and cognitively stimulating environments compared to parents who had always been in a low SES.

To our knowledge, only two studies have tested associations between parents’ social mobility and parenting, finding that upwardly mobile parents reported more cognitive stimulation and involvement in their children's schooling, relative to parents who were in a lower SES (Roksa & Potter, 2011), and that they differ in child-rearing values, encouraging thriftiness and feelings of responsibility in their children more than parents from either stable-low or stable-high socioeconomic backgrounds (Sieben, 2017). While this work offers valuable insights, it also has limitations, such as measuring parenting only via self-report or assessing child-rearing values rather than observed parenting practices, focusing only on mothers, and not testing whether pre-existing differences between parents who differ in social mobility explain differences in parenting. Our work sought to address these limitations by testing associations between social mobility and observed rather than self-reported parenting behaviors, in both mothers and fathers, and using a prospective longitudinal design to account for pre-existing different characteristics of parents, including in the parenting that parents themselves received when young, and in childhood cognition and self-control, all of which predict social mobility and parenting (J. Belsky et al., 2005; Forrest et al., 2011; Moffitt et al., 2011; Wertz et al., 2019).

Toward these ends, we used prospective, multigenerational data from the Dunedin Multidisciplinary Health and Development Study, a population-representative birth cohort, whose members were followed as they became parents. Data on parents’ own developmental histories were collected from childhood onwards, minimizing the risk of retrospective recall bias if parents are asked to report on their childhood SES (Warren et al., 2022) and other aspects of their childhood family environment (Bell & Bell, 2018), and making it possible to control for differences in characteristics and experiences that precede social mobility and parenting. Parents were observed interacting with their three-year-old child, allowing us to test the association between social mobility and parenting at a point in development when sensitive and cognitively stimulating parenting has been shown to affect some aspects of early cognition (Prime et al., 2023) and to initiate developmental cascades that promote prosocial behavior across childhood and adolescence (Malti & Speidel, 2023).

Using this data, we tested three hypotheses. First, we tested whether upwardly mobile parents provided more sensitive parenting and cognitively stimulating environments compared to parents who remained in a stable-low SES background, as would be predicted by economic resources and sociocultural models, and whether upwardly mobile parents provided less sensitive parenting and cognitively stimulating environments compared to parents who had always been in a stable-high SES background, as would be predicted by life-course perspectives on parenting. Second, we tested whether pre-existing differences in experienced parenting (in the family-of-origin) and in childhood characteristics (cognition and self-control) accounted for any observed differences in parenting behaviors between these groups. This tests whether mobility may impact parenting independently of early-life influences, or whether any apparent effects of upward mobility are attributable to parents’ pre-existing characteristics, a key threat to causal inference. Third, although our focus was primarily on upwardly mobile parents, we also investigated the parenting of parents whose circumstances meant that they had moved from a higher SES to a lower one (downwardly mobile), thus allowing us to test whether mobility in either direction had similar-sized associations with parenting.

## Method

### Sample

Participants (*N* = 1,037; 52% male) were members of the Dunedin Multidisciplinary Health and Development Study, a longitudinal investigation of health and behavior in a birth cohort. To be eligible for inclusion, participants had to be living in the greater Dunedin Metropolitan area 3 years after their birth (between April 1972 and March 1973) at Queen Mary Maternity Hospital, the only maternity hospital in Dunedin at the time. The 9% who declined or were unable to participate were no different from the 91% who agreed to take part in terms of maternal prenatal complications, birthweight, neonatal complications, or family SES (Poulton et al., 2015). Full details about the sample are reported elsewhere (Poulton et al., 2015). Dunedin cohort members are primarily of New Zealand European ethnicity; 8.6% reported Māori ethnicity at age 45. The cohort represented the full range of SES in the general population of New Zealand's South Island. On adult health, the cohort matches the New Zealand National Health and Nutrition Surveys on key health indicators (e.g., body mass index, smoking, visits to the doctor). Assessments with Dunedin cohort members were carried out at birth and ages 3, 5, 7, 9, 11, 13, 15, 18, 21, 26, 32, 38, and 45 years. Participation rates were well above 90% in all but one of the assessments (Bourassa et al., 2023; Poulton et al., 2015). All assessment phases of the Dunedin Study have received approval from the appropriate ethics committees. Written informed consent was obtained from all participants.

### The parenting study

In 1994, when Dunedin cohort members were 21–22 years old, a study of their parenting behavior was initiated (Parenting Study; J. Belsky et al., 2005). Participants were eligible if they had actively parented a child under 5 years old (the target recruitment age was age 3 years, but parents were still invited to participate if this age had passed, up to age 5 years). Parents (52% mothers; 48% fathers) and their children (50% female; 50% male) were visited in their home by an interviewer who conducted systematic observations of the home environment and who videotaped the parents interacting with their child in a series of tasks (described below). Children were observed when they were, on average, 3.4 years old, with 58% seen within 2 months of their third birthday (*SD* = 0.5 years; range 2.1–6.8 years). Parents were, on average, 32.7 years old at the time of the assessment (*SD* = 6 years; range 21.5–46.4 years). For the majority of parents, the child with whom they participated in the study was their first-born (91%) biological (97%) child. 9.2% of parents reported Māori ethnicity, comparable to the larger Dunedin study. All dyad pairs (i.e., mother/son, mother/daughter, father/son, father/daughter) were approximately equally represented. Study parents were paid NZ$40 as compensation.

**Figure 1:**
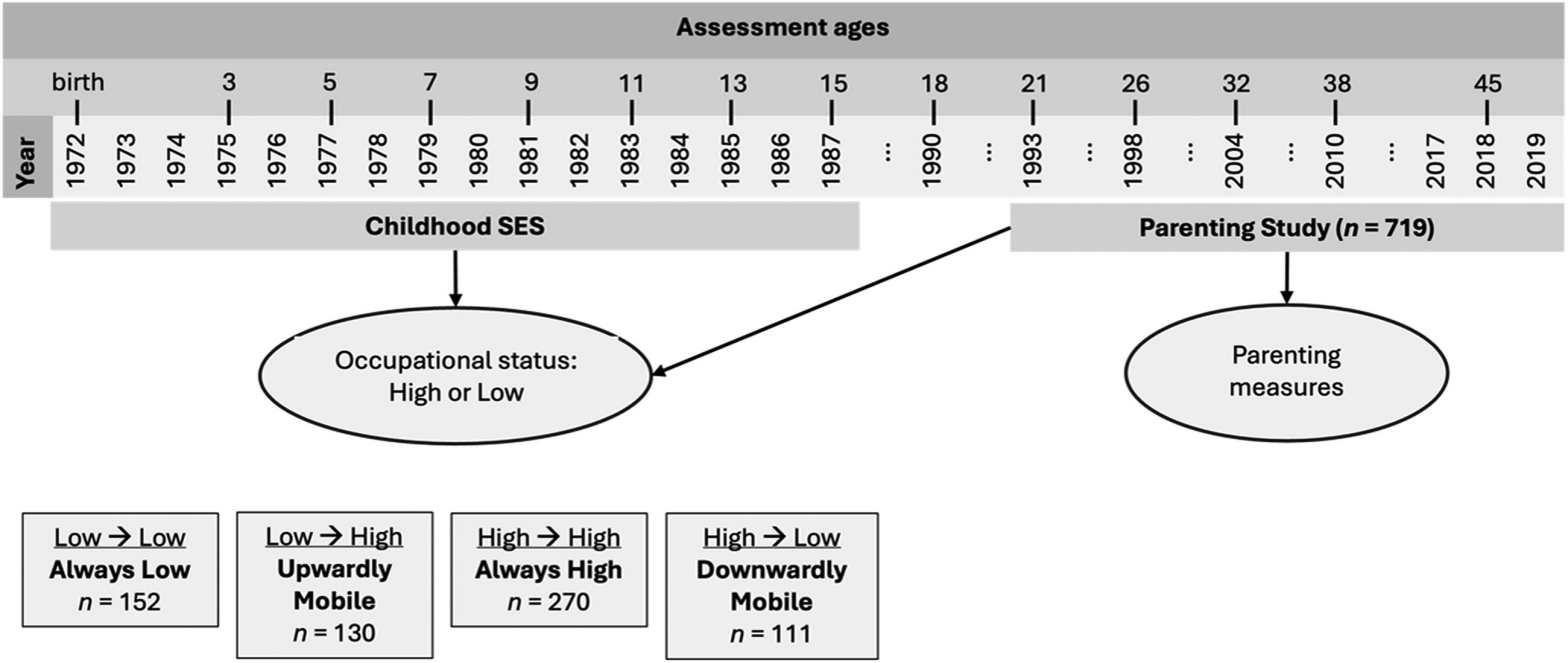
Study timeline. *Note:* The figure depicts when the data used in this study were collected, and how these were used to derive social mobility groups based on occupational status.

Over the time the Parenting Study ran, from 1994 when Dunedin cohort members were 21–22 years old through to 2019, when they were 46–47 years old, 719 had participated in the Parenting Study. This is out of 753 Dunedin cohort members who had been identified as eligible for participation, based on the above criteria, representing a participation rate of 95%. Analyses using sex-­adjusted regression analyses showed that Dunedin cohort members eligible for the Parenting study did not significantly differ from ineligible Dunedin cohort members in key characteristics, including childhood SES, childhood cognitive ability, and childhood self-control skills. Analyses using sex-adjusted regression analyses showed that eligible parents who agreed to participate in the Parenting Study had higher cognitive ability and self-control skills compared to eligible parents who declined, however, there were no differences between these groups in childhood SES, suggesting that our study population is broadly representative of the original cohort (which was population-representative) in terms of their early socioeconomic circumstances. A depiction of the timeline of our study is provided in Figure 1.

### Measuring parental social mobility as indexed by occupational status

*Childhood SES.* For our study parents, childhood SES was measured as previously described (Poulton et al., 2002), using a six-point scale assessing their parents’ self-reported occupational status during childhood (Elley & Irving, 1976). The scale places each occupation into one of six categories (1 = unskilled labourer, 6 = professional) based upon the educational level and income associated with that occupation in data from the New Zealand census, the NZ Standard Classification of Occupations at that time. The childhood SES of our study parents is the average of the highest SES level of either of their parents, assessed repeatedly at birth and at ages 3, 5, 7, 9, 11, 13, and 15 years. Data on childhood SES were available for *n* = 717 out of *n* = 719 study parents. We used a measure of average SES across multiple ages following previous research (Poulton et al., 2002) because measuring SES at a single point early in life does not capture stable exposure to low SES, given variation in SES across ages; for example, SES at birth and age 15 were correlated at r = 0.5. The cross-age variable of childhood SES thus better reflects the socioeconomic circumstances experienced by study parents while they grew up.

*Parent SES.* SES for study parents at the time of the parenting assessment was assessed using the same six-point scale, coded per the NZ Standard Classification of Occupations at that time. One study parent had missing data in this variable. For currently unemployed study parents (*n* = 22), occupational status was coded based on the last known occupation. Study parents who were homemakers at the time of the parenting assessment and who did not have a previous occupation (*n* = 54) were not included in the analyses, resulting in *n* = 663 with available SES data at both time points. However, we conducted sensitivity analyses in which we approximated homemakers’ occupational status using their reported educational level; this did not change the results (Table S1). Occupational status was only available for the study parent, not their partner.

To measure social mobility, we first constructed binary measures for study parents’ childhood and adult SES as indexed by occupational status, coding scores of 1–3 as lower SES and scores of 4–6 as higher SES. We then derived four social mobility groups corresponding to changes in and/or stability across occupational status from study parents’ childhood to parenthood: 1) stable low SES (*n* = 152; 22.9%, 2) upward mobility (*n* = 130; 19.6%, 3) downward mobility (*n* = 111; 16.7%, and 4) stable high SES (*n* = 270; 40.7%). The overall pattern of high SES stability, particularly for high-SES families, together with somewhat higher rates of upward vs downward mobility is consistent with findings from other countries such as the UK and US (Houle, 2011; Miller et al., 2020; Noble, 2000) and likely reflects both labour market trends at the time (e.g., an expansion of professional and managerial positions), and our binary coding of SES (which counts short-range changes in occupational status as stability). We used a categorical measure of mobility to align with previous research on social mobility, which typically conceptualises and measures mobility as movement between class positions rather than treating mobility as a continuous scale (Islam & Jaffee, 2024; Marshall & Firth, 1999). This approach also makes the direction and type of mobility clearer. However, we also conducted sensitivity analyses using continuous measures of SES to account for subtle SES differences between groups. For example, even though upwardly mobile parents and parents from stable-low SES backgrounds were both categorized as having experienced a low childhood SES, parents from stable-low SES backgrounds may have experienced a lower childhood SES to begin with. This could bias our models, which assume that these groups are comparable in their childhood SES. We tested this by comparing the groups using the continuous 6-point measures of SES. Upwardly mobile parents did not statistically significantly differ in childhood SES from parents from stable-low SES backgrounds (2.81 vs. 2.68; *t* = 1.77, *p* = .07), supporting the assumption that these groups came from comparable childhood socioeconomic circumstances. However, upwardly mobile parents’ SES at the time of the parenting assessment was not as high as that of parents from stable-high SES backgrounds (4.60 vs. 4.87; *t* = 3.23, *p* < .01). Downwardly mobile parents already had a lower SES in childhood compared to parents from stable-high SES backgrounds (4.30 vs. 4.60; *t* = 3.41, *p* < .01). Furthermore, their adult SES was higher than that of parents from stable-low SES backgrounds (2.40 vs. 2.13, *t* = 2.61, *p* < .01). To consider these differences in childhood and adult SES, we conducted sensitivity analyses in which we controlled for the continuous measures of childhood and adult SES when comparing groups (Table S2).

### Measuring parenting

Parenting measures for study parents are based on video observations of parent-child interactions and interviewer impressions of the home environment, as previously described (J. Belsky et al., 2005; Wertz et al., 2019). Briefly, for the video observations, each parent–child dyad was videotaped during the home visit in three increasingly challenging semi-structured situations, each lasting 10 minutes. The first situation involved free play, with the participants instructed to engage with their child using a varied set of age-appropriate toys that were provided. The second was a competing-task situation which involved the parent sitting on a chair while completing a (sham) questionnaire, while instructed not to permit the child to engage with a second set of attractive toys that was clearly (and purposefully) visible nearby, while the child had access to only one soft toy. The third task was a teaching task and involved the parent and child seated together, with the parent asked to provide whatever assistance the child needed to complete a set of activities that had been provided (Belsky et al., 2005). Each situation was rated by trained coders using a set of 7-point scales developed for the National Institute of Child Health and Human Development (NICHD) Study of Early Child Care (National Institute of Child Health & Human Development Early Child Care Research Network, 1999; NICHD Early Child Care Research Network, 2002). Scores for each scale were summed across the episodes to create across-episode total scores. To assess inter-coder reliability, 15% of the videotapes were randomly selected and coded by a second coder blind to all other information about the families. Inter-rater agreement ranged from 77 to 96 across ratings. The validity of these measurements is supported by NICHD Study findings linking individual differences in parenting with children's cognitive-linguistic and socio-emotional functioning (National Institute of Child Health & Human Development Early Child Care Research Network, 1999; NICHD Early Child Care Research Network, 2002). Following prior research (Wertz et al., 2019), we examined warm, sensitive parenting (“sensitive parenting”), indexed by an average across the subscales measuring “sensitive responsiveness” and “positive regard for the child” and reverse-coded “negative regard for the child”, “intrusiveness/over-control”, and “detachment/ disengagement” and cognitive stimulation, indexed by the “stimulation of cognitive development” subscale. Video observations of sensitive parenting and cognitive stimulation were correlated with each other, *r* = .61.

For the interviewer impressions of the caregiving environment, interviewers provided ratings after the home visit, using the Infant/Toddler Home Observation for Measurement of the Environment (HOME; Caldwell & Bradley, 1984). The HOME measures the quality and quantity of stimulation and support available to the child in the home environment (R. H. Bradley et al., 1988). Home interviewers indicated the absence (0) or presence (1) of each of 45 items pertaining to features of the home and family environment. To parallel the video assessment and follow prior work in this cohort (Wertz et al., 2019) we constructed bespoke measures of sensitive (*α* = .65) and cognitively-stimulating (*α* = .77) home environments. The sensitive subscale was an average score across 14 items from the HOME, reflecting parental expressions of affection and sensitivity (example items: “Parent's voice conveys positive feelings towards child”; “Parent does not express overt annoyance with or hostility to child”) (Mean = 0.88, SD = 0.12, Range 0.14–1). The cognitive stimulation subscale was an average score across 21 items from the HOME, reflecting the availability of learning materials and direct attempts by parents to teach skills and concepts (example items: “Parent provides toys that challenge child to develop new skills” and “Child has three or more books of his or her own”) (Mean = 0.87, SD = .13, Range 0.33–1). These two subscales were correlated with each other, *r* = .42. Both scales were z-standardized prior to analysis.

Video and HOME observations of sensitive parenting were correlated, *r* = .45, and this was true for video and HOME observations of cognitive stimulation measures as well, *r* = .41. To reduce issues of multiple testing and following previous research (Wertz et al., 2019), we constructed two separate, multi-method composite measures, one of sensitive parenting (*n* = 649) and the other of cognitive stimulation (*n* = 651), across video and HOME observations. We report results separately for each assessment method in the Supplement (Table S3).

### Measuring study parents’ childhood experiences and characteristics

The parenting experienced by study parents when they were young (sensitive parenting and cognitive stimulation) was measured during their early childhood (ages 3 and 5 years) and middle childhood (ages 7 and 9 years) and early adolescence (ages 13 and 15 years) using a combination of measures as previously reported (Belsky et al., 2005; Wertz et al., 2019). Briefly, experienced sensitive parenting was assessed at study parent ages 3 and 5 years using the Parental Attitude Research Instrument (PARI; (Schaefer et al., 1958); at ages 7 and 9 years using an interview with study parents’ mothers assessing their discipline practices, and three subscales of the Family Relations Index of the Family Environment Scales (FES; (Moos & Moos, 1981); and at ages 13 and 15, using the same three-subscales as at ages 7 and 9 years, along with a 24-item shortened version of Armsden and Greenberg's (1987) 53-item Inventory of Parent and Peer Attachment assessing young people's self-reported attachment to their parents. Cognitive stimulation was assessed at ages 3 and 5 years and 7 and9 years using study parent mothers’ reports on the activities and experiences of the child at home (such as being read to; dressing up) and away from home (such as farm, train, beach).

Study parents’ cognitive ability in childhood was assessed at ages 7, 9, 11, and 13 years, using the Wechsler Intelligence Scale for Children—Revised (WISC-R; (Wechsler, 1974). Scores were averaged across age and standardized to *M* = 100, *SD* = 15. Study parents’ childhood self-control was measured using multiple measures as previously described (Moffitt et al., 2011; Richmond-Rakerd et al., 2021); including observational ratings of participants’ lack of control (ages 3 and 5) and parent, teacher, and self-reports of impulsive aggression, overactivity, lack of persistence, inattention, and impulsivity (ages 5, 7, 9, and 11). We used a measure that had been constructed in previous research (Moffitt et al., 2011); based on principal components analysis the standardized measures were averaged into a single composite score (*M* = 0, *SD* = 1), and reverse-coded so that a high score reflects high self-control.

### Analytic strategy

To test whether upwardly mobile parents provided more sensitive parenting and cognitively-stimulating environments than parents from stable-low and stable-high SES backgrounds, we used one-way analyses of covariance (ANCOVAs) followed by planned contrasts comparing upwardly mobile to stable-low SES parents, and upwardly mobile to stable-high SES parents. We adjusted for parent age because study parents had their children at different ages (M = 29.6 years; SD = 6, Range 15–43), and age at parenthood is associated with social mobility (Johansen et al., 2020) as well as with parenting, including in this cohort (Wertz et al., 2019). We additionally adjusted for the child's age in months (M = 40.3, SD = 6.5, Range 25–81), as well as the sex of the study parent (52.3% female) and their child (50% female) (see Table S4 for descriptives for each group separately), which may be associated with mobility and parenting. We were underpowered to formally test interactions with parent sex, but report analyses separately for mothers and fathers in Table S5. To test whether any parenting differences across mobility groups were accounted for by pre-existing differences in study parents’ experienced parenting and childhood cognitive ability and self-control, we added these variables to the ANCOVA models. We controlled for study parents’ experienced sensitive parenting when predicting their sensitive parenting as the outcome, and for study parents’ experienced cognitive stimulation when predicting their cognitively-stimulating parenting as the outcome. To test whether downwardly mobile parents differed in their parenting from parents in stable-low and stable-high SES backgrounds, we used the same ANCOVA models as described above, followed by planned contrasts comparing downwardly mobile parents to parents from stable-high SES backgrounds, and downwardly mobile parents to parents from stable-low SES backgrounds.

We used an adjusted *p*-value of .013 instead of .050 to evaluate the results of the planned contrasts, due to the four comparisons in our analyses (i.e., upwardly mobile vs stable-high SES and stable-low SES; downwardly mobile vs stable-low SES and stable-high SES). Differences between social mobility groups in parenting are expressed as Cohen's *d*, to provide a standardized effect size for interpreting the difference between the group means. A commonly used interpretation is to refer to effect sizes as small (d = 0.2), medium (d = 0.5), and large (d = 0.8) based on benchmarks suggested by Cohen (1988).

Sample sizes varied slightly across analyses depending on available data; the specific sample size for each analysis is detailed in each Table and Figure. All analyses were pre-registered https://osf.io/m6geh and changes to the pre-registration are outlined in Table S6 using a standardized template (Willroth & Atherton, 2024). Deviations included (a) not examining educational attainment as a factor accounting for differences in parenting between social mobility groups due to concerns about multicollinearity between occupational status and education, and (b) adding sensitivity analyses to test whether differences in continuous measures of childhood or adult SES accounted for differences in parenting between mobility groups. All analyses were conducted in Stata version 18.5.

## Results

### Is parental upward mobility associated with differences in parenting?

**Figure 2:**
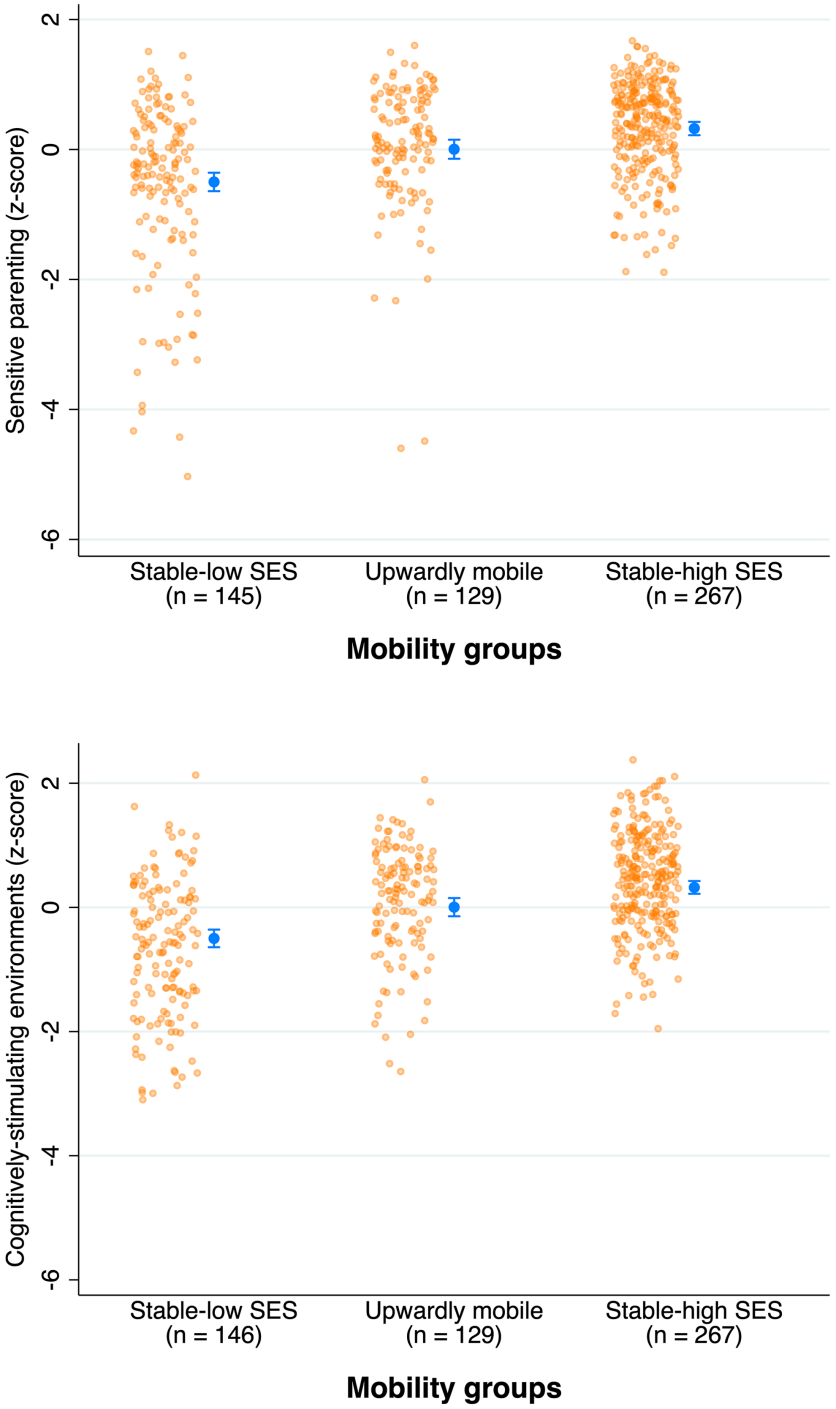
Mean parenting scores across social mobility groups. *Note*: The figure shows mean scores for sensitive parenting (upper panel) and cognitivelystimulating environments(lower panel) for parents in three social mobility groups in the Dunedin cohort: stable-low (i.e., having been in a low-SES background in childhood and at the time of the parenting assessment), upwardly mobile (i.e., having been in a low-SES background in childhood and high SES at the time of the parenting assessment), and stable-high (i.e., having been in a low-SES background in childhood and at the time of the parenting assessment). The orange dots show the distribution of the original data; each dot represents one participant. The n varied slightly across parenting measures; the exact *n* for each group is reported in the Figure. Error bars indicate standard errors.

We found evidence for overall differences across the social mobility groups in sensitive parenting, *F*(3, 641) = 17.5, *p* < .001, R² for full mode including covariatesl = 15.3% [6.5% for social mobility only], and cognitive stimulation, *F*(3, 643) = 27.0, *p* < .001, R² for full model including covariates = 30.7% [11.5% for social mobility only]. Consistent with our hypotheses, the group means (Figure 2) and planned contrasts (Table 1, Model 1) revealed that upwardly mobile parents provided, on average, more sensitive parenting (Cohen's d = .44 [95%CI .20; .68], *p* < .01) and cognitively stimulating environments (Cohen's d = .51 [95%CI .27; .75], *p* < .01) compared to parents who remained in a low SES from childhood to adulthood. However, upwardly mobile parents provided less sensitive parenting (Cohen's d = −.32 [95%CI −.54; −.11], *p* = .01) and less cognitively stimulating environments (Cohen's d = −.40 [95%CI −.62; −.19], *p* < .01) compared to parents who had always been in a high SES from childhood to adulthood. For cognitively stimulating environments, this finding remained after accounting for differences in achieved adult SES between these groups, using a continuous measure of SES (Table S1). However, for sensitive parenting, upwardly mobile parents no longer differed statistically significantly from parents from stable-high SES backgrounds after accounting for differences in achieved adult SES (Table S2). The findings were generally consistent when analyzing video and home observations of parenting separately (Table S3).

**Table 1:** Comparisons of upwardly mobile parents to parents from stable SES backgrounds.

Controlled for:	Model 1 (basic demographics)		Model 2 (early parenting)		Model 3 (childhood skills)	
	*d*	95% CI	*d*	95% CI	*d*	95% CI
Sensitive parenting						
*Upwardly mobile vs:*						
Stable low SES	0.44^a^	0.20, 0.67	0.38^a^	0.14, 0.62	0.31^a^	0.07, 0.54
Stable high SES	−0.32^a^	−0.54, −0.11	−0.28^b^	−0.49, −0.07	−0.17	−0.38, 0.04
Cognitively stimulating environments						
*Upwardly mobile vs:*						
Stable low SES	0.51^a^	0.27, 0.75	0.44^a^	0.20, 0.68	0.35^a^	0.11, 0.59
Stable high SES	−0.40^a^	−0.62, −0.19	−0.35^a^	−0.56, −0.30	−0.21	−0.42, 0.00

*Note.* The Table reports standardized mean differences (expressed as Cohen's d) in parenting environments and behaviors between parents in different social mobility groups. Model 1 accounted for study member and offspring age and sex, which were also included in all subsequent models. Model 2 accounted for either experienced sensitive parenting in childhood (if sensitive parenting was the outcome) or cognitively stimulating environments in childhood (ifcognitive stimulation was the outcome). Model 3 accounted for childhood cognition and self-control. The sample size for upwardly mobile parents was *n* = 129; for parents from stable-low SES backgrounds, *n* = 145 for sensitive parenting and *n* = 146 for cognitive stimulation; and for parents from stable-high SES backgrounds, *n* = 267.

^a^statistically significant after correcting for multiple testing (*p* < 0.0125).

^b^no longer statistically significant after correcting for multiple testing.

### Do parents’ childhood experiences of parenting account for parenting differences between social mobility groups?

When they were children, upwardly mobile participants had experienced more sensitive parenting and more cognitively stimulating environments compared to parents from stable-low SES backgrounds, and less compared to those from stable-high SES backgrounds (Table S7). Furthermore, parents who had experienced more sensitive parenting and cognitively stimulating environments tended to provide more such parenting and environments to their own children (Table S8). We next tested whether differences in experienced parenting accounted for differences in parenting across mobility groups. Adjusting for experienced parenting reduced differences between the groups by 13–15%. In adjusted models, upwardly mobile parents continued to provide more sensitive parenting and cognitively stimulating environments than parents who had remained in a low SES, and less cognitively stimulating environments than parents who had been in a high SES from childhood to adulthood. However, differences with parents from stable-high SES in sensitive parenting no longer reached statistical significance (Table 1, Model 2). This finding indicates that apparent differences in parenting between upwardly mobile parents and those from stable-high SES backgrounds are partly accounted for by pre-existing differences between these groups in experienced parenting.

### Do parents’ childhood cognitive and self-control skills account for parenting differences between social mobility groups?

As with experienced parenting, upwardly mobile parents differed in their childhood characteristics, having displayed greater cognitive (but not self-control) skills compared to those from stable-low SES backgrounds, and fewer compared to those from stable-high SES backgrounds (Table S7). Furthermore, parents who had displayed more cognitive and self-control skills in childhood provided more sensitive parenting and cognitively stimulating environments to their own children (Table S8). After accounting for childhood cognition and self-control, differences between mobility groups were reduced by approximately 30% for comparisons between upwardly mobile parents and those from stable-low SES backgrounds, and by approximately 50% for comparisons between upwardly mobile parents and those from stable-high SES backgrounds. In the adjusted models, upwardly mobile parents continued to provide more sensitive parenting and cognitively stimulating environments compared to parents who had remained in a low SES, but the difference from parents in a stable-high SES was smaller and no longer statistically significant (Table 1, Model 3). This finding indicates that apparent differences in parenting between upwardly mobile parents and those from stable-high backgrounds are partly accounted for by pre-­existing differences in childhood cognition and self-control.

### Is parental downward mobility associated with differences in parenting?

Comparing group means and planned contrasts for downwardly mobile parents revealed a pattern of findings that mirrored those for upwardly mobile parents (Table 2, Model 1). That is, downwardly mobile parents provided less sensitive parenting (Cohen's d = −.46 [95%CI −.68; −.23] and less cognitively stimulating environments (Cohen's d = −.54 [95%CI −.76; −.31], *p* < .01) compared to parents who had remained in a high SES from childhood through to adulthood. However, they provided more sensitive parenting (Cohen's d = .33 [95%CI.08; .58], *p* < .01) and more cognitively stimulating environments (Cohen's d = .36 [95%CI.11; .61], *p* < .01) compared to parents who had always been in a low SES. Differences between parents were not accounted for by the continuous measures of childhood or adulthood SES (Table S2).

**Table 2:** Comparisons of downwardly mobile parents to parents from stable SES backgrounds.

Controlled for	Model 1 (basic demographics)		Model 2 (early parenting)		Model 3 (childhood skills)	
	*d*	95% CI	*d*	95% CI	*d*	95% CI
Sensitive parenting						
*Downwardly mobile vs:*						
Stable high SES	−0.46^a^	−0.68, −0.23	−0.41^a^	−0.64, −0.19	−0.31^b^	−0.53, −0.09
Stable low SES	0.33^a^	0.08, 0.58	0.28	0.03, 0.53	0.20	0.05, 0.45
Cognitively stimulating environments						
*Downwardly mobile vs:*						
Stable high SES	−0.54^a^	−0.76, −0.31	−0.50^a^	−0.72, −0.27	−0.37^a^	−0.59, −0.14
Stable low SES	0.36^a^	0.11, 0.61	0.30^a^	0.05, 0.55	0.20	−0.05, 0.44

*Note.* The Table reports standardized mean differences (expressed as Cohen's d) in parenting environments and parenting behaviors between parents in different social mobility groups. Model 1 accounted for study member and offspring age and sex, which were also included in all subsequent models. Model 2 accounted for either experienced sensitive parenting in childhood (if sensitive parenting was the outcome) or cognitively stimulating environments in childhood (ifcognitive stimulation was the outcome). Model 3 accounted for childhood cognition and self-control. The sample size for downwardly mobile parents was *n* = 109; for parents from stable-high SES backgrounds, *n* = 267, and for parents from stable-low backgrounds, *n* = 145 for sensitive parenting and *n* = 146 for cognitively stimulating environments.

^a^statistically significant after correcting for multiple testing (*p* < 0.0125).

^b^no longer statistically significant after correcting for multiple testing.

As before, we accounted for pre-existing differences in experienced parenting and childhood characteristics in these analyses. After accounting for pre-existing differences, downwardly mobile parents more closely resembled parents from stable-low SES backgrounds than parents from stable-high SES backgrounds in their provision of cognitively stimulating environments and sensitive parenting (Table 1, Models 2 and 3).

## Discussion

Previous research has documented the impact of SES on parenting and family environments and raised the question of whether improved family socioeconomic circumstances may lead to the provision of more stimulating environments and parenting. Here, an unusually rich data set provided a unique opportunity to address this question. The study, extending from childhood through adulthood, included prospective assessments of SES as indicated by occupational status across two generations as well as measures of individual characteristics and experienced parenting that predated the transition to parenthood. In addition, the study included detailed observations of mothers and fathers interacting with their 3-year old children, to test hypotheses about whether changes in occupational status from childhood to adulthood predicted parents’ ability to provide more sensitive parenting and cognitively-stimulating environments, two key dimensions repeatedly linked to children's cognitive, educational, and socioemotional outcomes (Madigan et al., 2019; Pinquart, 2016, 2017).

Our analyses revealed three key findings. First, parents who had been able to move from a lower SES in childhood to a higher SES by the time they were parents—i.e., who were upwardly mobile—provided more sensitive parenting and cognitively stimulating environments than parents who remained in a low SES into adulthood, even after accounting for a number of pre-existing differences between these parents and the age of parenthood. This finding suggests that even though differences in childhood experiences and circumstances lead individuals onto different mobility trajectories, improvements in SES, as indicated by occupational status, are associated with parenting outcomes over and above these pre-existing differences.

Second, despite upwardly mobile parents providing more sensitive parenting and cognitively stimulating environments, there remained apparent differences between these parents and parents with stable-high SES. However, these differences were entirely explained by parents’ childhood experiences and characteristics. That is, as children, upwardly mobile individuals tended to have lower cognitive and self-control skills than children from stable-high SES backgrounds, which explained why they differed in parenting their own children years later. This finding highlights the role of developmental histories for understanding parenting differences, consistent with life-course perspectives on parenting (J. Belsky, 1984; J. Belsky & Jaffee, 2006).

Third, findings for downwardly mobile parents mirrored those for upwardly mobile parents, such that parents who went from a higher SES in childhood to a lower SES in adulthood more closely resembled the parents with whom they shared an adult SES. Downward mobility is relatively rarely studied compared to upward mobility, despite research suggesting that moving from a higher SES in childhood to a lower SES in adulthood has become more common for recently born generations in several countries (Li & Devine, 2011; Song et al., 2020). Our findings corroborate the conclusions for upwardly mobile parents and suggest that social mobility in either direction may be consequential for parenting, and to a similar degree.

### Limitations

Our study has limitations. First, it is observational and thus cannot establish causal associations. Despite the prospective-­longitudinal design and control for pre-existing differences between mobility groups, there may be additional unobserved variables, such as personal characteristics or genetic influences on social mobility and parenting (D. W. Belsky et al., 2018; Wertz et al., 2019). To the extent that these are not captured by our control for early experiences and characteristics, they may influence or account for some of the associations we observe. Similarly, contextual factors such as cultural influences (Lansford, 2022), economic climate (Doepke et al., 2019), and relationship characteristics (Yu & Gamble, 2008) may also play a role. Furthermore, there were some differences in initial and achieved SES between mobility groups, and even though we controlled for these in sensitivity analyses, they may point to other unobserved differences, e.g., in wealth. Causally informative designs, such as natural experiments that examine the impact of changes in socioeconomic circumstances, are needed to infer causal effects (Akee et al., 2018). Second, parents had children at different ages, and having a child at an earlier age is associated with lower educational attainment and earnings that decrease the chances of upward mobility (Johansen et al., 2020). Furthermore, parents of different ages raised their children in different social and economic contexts; the parents who made the earliest transition to parenthood were raising toddlers in the 1990s, whereas those who made a later transition to parenthood were raising toddlers in the 2010s. Income inequality increased over the study period (OECD, 2023), which may affect parenting (Doepke et al., 2019), and there have also been shifts in the “culture of parenting”, with increased emphasis on sensitive and cognitively stimulating parenting. Although we statistically controlled for differences in parental age, which partially captures these effects, replication in samples with a more homogenous age group of parents is needed. Third, concerns have been raised that widely used measures of parenting, including those used in this study, are shaped by assumptions aligning more closely with middle-class norms and values, which could limit their ability to fully capture the diversity of parenting practices across different socioeconomic contexts (Dermott & Pomati, 2016). As a result, our findings may overlook parenting strategies that are effective and responsive to the specific challenges and demands faced by lower-SES families. Fourth, our findings were for occupational status, and they do not necessarily generalize to other indicators of SES, such as income or education, which tend to be highly correlated but also show differences in associations with outcomes, including parenting (Davis-Kean, 2005). The NZ classification of occupational status is based on the income and education associated with occupations, so this information is incorporated in our analyses, however due to data constraints we were unable to test the effects of mobility in each of the three indicators separately, as well as that of other economic indicators such as wealth, or mobility and volatility in income which was previously shown to be associated with parenting (Hill et al., 2013). Another relevant measure is educational attainment, given policy efforts in many countries in recent decades to widen educational access and increase educational mobility. Future studies that follow more recent generations into parenthood are needed to test the impact of mobility on these indicators. Fifth, group differences in parenting were modest, and distributions overlapped, reflecting variability in parenting behaviors in each group. The modest effect sizes and variability are unsurprising, considering that parenting is multiply determined, with parents’ social mobility representing only one of many factors. Nonetheless, social mobility explained a meaningful proportion of variance in parenting outcomes, particularly in cognitively stimulating environments, and, even though effects were modest, they were robust to controlling for a variety of confounding factors. Sixth, we had very little information about parents’ partners, such as their social mobility, parenting, and personal characteristics, which are likely to contribute to variation in parenting, and should be taken into consideration in future research. Finally, rates of social mobility and socioeconomic inequality vary across countries (Causa & Johansson, 2010), and findings may thus not generalize to parents from countries with different SES distributions or opportunities for mobility.

### Implications for research and policy

The findings of this research have implications for research on the origins of parenting differences. The finding that parents with similar SES backgrounds in childhood differed in their parenting depending on their adult SES, even after accounting for other differences associated with SES in childhood, suggests the conclusion that current SES is a stronger predictor of parenting than childhood SES. This apparent importance of parents’ current SES is perhaps unsurprising, as higher SES allows parents to draw on economic resources that support parenting, and may also lead them to adopt different parenting norms and expectations, consistent with the “economic resources” and “sociocultural” pathways we lay out. However, the comparison of parents with a similar SES in adulthood—that is, upwardly mobile compared to stable-high SES and downwardly mobile compared to stable-low—complicates this conclusion, because it suggest small lingering effects of childhood experiences, even if these effects were more due to childhood skills rather than childhood economic status. Thus, although the findings support the importance of current SES for parenting, consistent with theory and prior research, they also highlight some heterogeneity in parenting even among parents with similar adult SES, due to childhood experiences and circumstances. These observations underscore the importance of considering parents’ experiences and exposures from before they become parents when studying parenting (J. Belsky, 1984; Kretschmer, 2021).

Our results also have implications for policy. First, reducing socioeconomic inequities and increasing intergenerational social mobility has been an explicit goal of government policy in several countries. Our findings suggest that in addition to improving individuals’ own health, wealth, and wellbeing as shown by previous research, upward social mobility may also have intergenerational impacts through differences in parenting practices. Second, the finding that parents with a lower SES in adulthood—due to downward mobility or because they had remained in a low SES from childhood to adulthood—provided less sensitive parenting and cognitively stimulating environments underscores the need to address the social-structural factors that make it both challenging and, in some cases, counterproductive for lower-income families to use those parenting practices (Lareau, 2002). Third, our findings call for greater attention to downward mobility and its impacts on families intergenerationally. This is particularly relevant given that downward mobility appears to have increased for younger generations (Li & Devine, 2011; Song et al., 2020), which is thought to be partly due to the contraction of middle class jobs and, particularly in the US, the escalating costs of post-secondary education and health care. Lastly, our findings highlight how parenting is shaped by early-childhood characteristics such as self-control and cognitive skills, in addition to parents’ current circumstances such as their SES. Early childhood programs that target these skills have already shown to predict better health, labor outcomes and reduced crime for program participants (Barr & Gibbs, 2022; García & Heckman, 2023). Our findings suggest that, by modifying these skills, these programs could also affect parenting once program participants have their own children.

Taken together, our findings show how movements up (and down) the socioeconomic ladder are reflected in the parenting provided to young children, and they suggest implications for research—including a greater consideration of the life-course determinants of parenting behaviors—and for policy, including supporting economically disadvantaged families, preventing downward mobility, and investing in children's early development, thereby contributing to closing socioeconomic inequities between families and affecting successive generations of children.

## Supplementary Material

aacaf050_Supplementary_Data

## Data Availability

The data necessary to reproduce the analyses presented here are not publicly accessible due to the confidentiality of participants who are part of an ongoing life-course study. Access to Dunedin cohort data is subject to managed data access procedures (see: https://dunedinstudy.otago.ac.nz/for-investigators/policy-statement-code-of-practice). The analytic code necessary to reproduce the analyses presented in this paper is publicly accessible. Code is available from the corresponding author (JW). Not all of the materials necessary to attempt to replicate the findings presented here are publicly accessible. Materials are available from the corresponding author (JW).

## Statement of preregistration

The analyses presented here were preregistered. The preregistration is available at the following URL: https://osf.io/m6geh, and changes to the pre-registration are outlined in Table S6 using a standardized template (Willroth & Atherton, 2024).
