# Supplementary material for: Social mobility and parenting: Testing associations in a prospective longitudinal cohort study
Source: Child Dev. 2026 Feb 20;97(2):585–97. doi: 10.1093/chidev/aacaf050 (PMC13046069; doi:10.1093/chidev/aacaf050)
Supplement: aacaf050_Supplementary_Data [file aacaf050_supplementary_data.docx]

**Supplemental Material**

## Table S1. Parenting differences when including homemakers in the analysis.

|  | |  | | | | |
| --- | --- | --- | --- | --- | --- | --- |
| Outcome: | Sensitive Parenting | | Cognitively stimulating environments | |  |  |
| *Upwardly mobile compared to:* | | | |  | |  |
| Stable low SES | 0.43*  (0.20; 0.66) | | 0.47*  (0.24; 0.70) | |  |  |
| Stable high SES | -0.29^  (-0.49; -0.09) | | -0.43*  (-0.64; -0.22) | |  |  |
| *Downwardly mobile compared to:* | | | |  | |  |
| Stable high SES | -0.44*  (-0.65; -0.22) | | -0.54*  (-0.75; -0.32) | |  |  |
| Stable low SES | 0.30*  (.06; .54) | | 0.35*  (.11; .59) | |  |  |

*Note:* The Table reports standardized mean differences (expressed as Cohen’s d) in parenting measures between parents in different social mobility groups, when including homemakers (n=54) who had been excluded in our primary analyses due to missing data in occupational status at the time of the parenting assessments. Occupational status data was approximated using information on participants’ self-reported educational attainment following the imputation procedure set out in the New Zealand Socio-economic Index 1996 (Statistics New Zealand, 2003) so that, on the six-point occupational status measure, a PhD, JD or MD corresponds to a value of 6; a MA or BA corresponds to a value of 5; a Sixth Form Certificate or School certificate corresponds to a value of 4; and an overseas school qualification, being a current student, or no school certificate corresponds to a value of 3 [note: because occupational status reflects more than qualifications alone, ‘no school certificate’ does not automatically correspond to the lowest occupational status in this imputed categorisation].

^no longer significant after correcting for multiple testing

*statistically significant after correcting for multiple testing (p=0.013)

## Table S2. Parenting differences between groups controlling for childhood and adult SES.

|  | | Controlled for Childhood SES | | | | | Controlled for Adult SES | | |
| --- | --- | --- | --- | --- | --- | --- | --- | --- | --- |
| Outcome: | Sensitive Parenting | | Cognitively stimulating environments | | Sensitive Parenting | | | Cognitively stimulating environments | |
| *Upwardly mobile compared to:* | | | |  | |  | | |  |
| Stable low SES | 0.43*  (0.19; 0.67) | | 0.50*  (0.26; 0.74) | | - | | | - | |
| Stable high SES | - | | - | | -0.27^^^  (-0.49; -0.06) | | | -0.31*  (-0.52; -0.10) | |
| *Downwardly mobile compared to:* | | | |  | |  | | |  |
| Stable high SES | -0.44*  (-0.67; -0.21) | | -0.51*  (-0.74; -0.29) | | - | | | - | |
| Stable low SES | - | | - | | 0.30*  (0.05; 0.55) | | | 0.30*  (0.50; 0.54) | |

*Note:* The Table reports standardized mean differences (expressed as Cohen’s d) in parenting behaviours between parents in different social mobility groups, controlling for the continuous score of childhood or adult SES (in addition to adjustment for study member and offspring age and sex). Outcomes represent composite measures of parenting derived from HOME and video observations.

^no longer significant after correcting for multiple testing

*statistically significant after correcting for multiple testing (p=0.0125)

##

## Table S3. Parenting differences between groups separately for video and home observations.

|  | | Video observations | | | | | HOME observations | | |
| --- | --- | --- | --- | --- | --- | --- | --- | --- | --- |
| Outcome: | Sensitive Parenting | | Cognitively stimulating environments | | Sensitive Parenting | | | Cognitively stimulating environments | |
| *Upwardly mobile compared to:* | | | |  | |  | | |  |
| Stable low SES | 0.36*  (0.12; 0.60) | | 0.28^  (0.04; 0.52) | | 0.39*  (0.15; 0.63) | | | 0.57*  (0.32; 0.81) | |
| Stable high SES | -0.31*  (-0.52; -0.10) | | -0.32*  (-0.53; -0.11) | | -0.22^  (-0.43; -0.01) | | | -0.40*  (-0.62; -0.19) | |
| *Downwardly mobile compared to:* | | | |  | |  | | |  |
| Stable high SES | -0.25^  (-0.47; -0.02) | | -0.39*  (-0.61; -0.16) | | -0.48*  (-0.71; -0.26) | | | -0.50*  (-0.72; -0.27) | |
| Stable low SES | 0.41*  (0.15; 0.66) | | 0.22  (-0.03; 0.48) | | 0.17  (-0.08; 0.42) | | | 0.37*  (0.12; 0.62) | |

*Note:* Table reports standardized mean differences (expressed as Cohen’s *d*) separately for parenting assessed using video and HOME observations. Models are adjusted for study member and offspring age and sex.

*statistically significant after correcting for multiple testing (p<0.0125)

^no longer statistically significant after correcting for multiple testing

## Table S4. Descriptive information on parents in the different mobility groups.

|  | Stable-lowSES | Upwardlymobile | Downwardlymobile | Stable-high SES |
| --- | --- | --- | --- | --- |
| Parent sex (female) | 51% | 49% | 47% | 50% |
| Child sex (female) | 47% | 51% | 56% | 47% |
|  | M (SD) | M (SD) | M (SD) | M (SD) |
| Age at birth of child | 26.7 (5.5) | 32.3 (4.7) | 27.5 (6.1) | 32.2 (4.7) |
| Childhood SES | 2.6 (0.6) | 2.7 (0.5) | 4.4 (0.7) | 4.7 (0.8) |
| Parent SES | 2.1 (0.8) | 4.6 (0.8) | 2.4 (0.8) | 4.9 (.08) |
| Child age (months) | 41.0 (7.6) | 39.7 (5.5) | 40.2 (7.0) | 39.9 (5.0) |

## *Note:* Descriptives are reported for the n=663 participants with available data in child and parent SES. The group sizes were as follows: stable-low SES, n=152; upwardly mobile, n=130; downwardly mobile, n=111, and stable-high SES, n=270. Table S5. Comparisons of social mobility groups separately for mothers and fathers.

|  | Combined | Mothers | Fathers |
| --- | --- | --- | --- |
| Sensitive parenting | *d* (95% CI) | *d* (95% CI) | *d* (95% CI) |
| Up vs low | .44* ( .20, .67) | .47* (.13, .81) | .37^ (.03, .71) |
| Up vs high | -.32* (-.54, .11) | -.22 (-.52, -.08) | -.47^ (-.77, -.17) |
| Down vs high | -.46* (-.68, .23) | -.18 (-.40, .04) | -.65* (-.96, -.33) |
| Down vs low | .33* ( .08, .58) | .56* (.30, .81) | .17 (-.18, .52) |
| Cognitively stimulating | Combined | Mothers | Fathers |
| environments | *d* (95% CI) | *d* (95% CI) | *d* (95% CI) |
| Up vs low | .51* (.27, .75) | .35^ (.01, .69) | .65* (.30, .99) |
| Up vs high | -.40* (-.62, .19) | -.48* (-.78, -.18) | -.32^ (-.62, -.02) |
| Down vs high | -.54* (-.76, .31) | -.34 (-.66, -.01) | -.74* (-1.05, .42) |
| Down vs low | .36* (.11, .61) | .44* (.08, .80) | .36^ (.01, .71) |

## *Note.* The Table reports standardized mean differences (expressed as Cohen’s d) in parenting environments and parenting behaviours between parents in different social mobility groups, separately for mothers, fathers, and mothers and fathers combined. Covariates are offspring age and sex, and parental age. Depending on parent sex and outcome variable, sample sizes ranged from n=71 to n=75 for always-low SES parents; n=64 to n=65 for upwardly mobile parents; 50 to 58 for downwardly mobile parents and n=132 to 135 for always-high SES parents.

*statistically significant after correcting for multiple testing (p<0.0125)

^no longer statistically significant after correcting for multiple testing

## Table S6. Preregistration deviations.

| **Deviations** | | | | | |
| --- | --- | --- | --- | --- | --- |
| # | Details | | Original Wording | Deviation Description | Reader Impact |
| 1 | Type | Research Q(s) | We originally planned to examine parents’ educational attainment as a potential factor accounting for differences in parenting practices between those in different social mobility groups. | Given the high correlations between adult occupational status and educational attainment, including in our sample, and given that the NZ SES classification is based on the education of census respondents who held an occupation, we were concerned about multicollinearity, as our social mobility groups were derived from adult occupational status. We thus did not test educational attainment as a factor that might account for differences in parenting between mobility groups. | Readers’ interpretation of the study and results should not be impacted by this change. |
|  | Reason | New knowledge |  |  |  |
|  | Timing | After data access |  |  |  |
| **Unregistered Steps** | | | | | |
| # | Details | | Original Wording | Unregistered Step Description | Reader Impact |
| 1 | Type | Analysis | This was unaddressed in the preregistration. | We tested whether potential differences in the continuous measure of childhood SES between groups who we assumed to have a similar childhood SES explained differences between their parenting practices, and we also tested whether differences in adult SES accounted for differences in parenting between groups that we assumed had a similar adult SES. | This additional analysis should make the reader more confident that our findings are robust to small differences in childhood and adult SES between groups. |
|  | Timing | After results known |  |  |  |
| # | Details | | Original Wording | Unregistered Step Description | Reader Impact |
| 2 | Type | Analysis | This was unaddressed in the preregistration. | In response to a reviewer comment, we tested whether our analyses changed when including homemakers in our analyses. In these analyses, we approximated homemakers’ occupational status using parents’ reported education. | This analysis should increase confidence in the robustness of our analyses, to small changes in sample composition. |
| 3 | Type | Analysis | This was unaddressed in the preregistration. | In response to a reviewer comment, we ran our models separately for mothers and fathers. | These analyses are not as robust as the analyses in the full sample because the numbers in the individual mobility groups become quite small. However, they may satisfy readers who wonder about sex differences, and they may stimulate future research in larger samples. |

## Table S7. Differences between mobility groups in childhood experienced parenting and cognition and self-control.

|  | Experienced sensitive parenting | | Experienced cognitively stimulating environments | | Childhood cognition | | Childhood self-control | |
| --- | --- | --- | --- | --- | --- | --- | --- | --- |
|  | *d* | 95% CI | *d* | 95% CI | *d* | 95% CI | *d* | 95% CI |
| *Upwardly mobile vs:* | |  |  |  |  |  |  |  |
| Stable low SES | 0.41* | 0.17; 0.64 | 0.42* | 0.19; 0.66 | 0.72* | 0.48; 0.97 | 0.23 | -0.01; 0.46 |
| Stable high SES | -0.34* | -0.55; -0.13 | -0.26* | -0.47; -0.05 | -0.58* | -0.80; -0.37 | -0.42* | -0.64; -0.21 |
| *Downwardly mobile vs:* | |  |  |  |  |  |  |  |
| Stable high SES | -0.29^ | -0.52; -0.07 | -0.19 | -0.41; 0.03 | -0.52* | -0.74; -0.29 | -0.36* | -0.58; -0.14 |
| Stable low SES | 0.48* | 0.23; 0.72 | 0.55* | 0.30; 0.80 | 0.75* | 0.49; 1 | 0.33* | 0.08; 0.57 |

*Note:* The Table reports standardized mean differences (expressed as Cohen’s d) in childhood experienced parenting (sensitive parenting and cognitively stimulating environments), cognition and self-control, between parents in different social mobility groups. All models are adjusted for study member age and sex.

*statistically significant after correcting for multiple testing (p<0.0125)

^no longer statistically significant after correcting for multiple testing

**Table S8. Associations between parents’ childhood experienced-parenting and childhood cognition and self-control with sensitive parenting and cognitively stimulating environments.**

|  | Outcome | |
| --- | --- | --- |
|  | Sensitive parenting | Cognitively stimulating environments |
| Predictor | β (95% CI) | β (95% CI) |
| Childhood experienced sensitive parenting | 0.19  (0.12; 0.27) | - |
| Childhood experienced cognitively stimulating environments | - | 0.23  (0.17; 0.30) |
| Childhood cognitive ability | 0.28  (0.21; 0.35) | 0.32  (0.26; 0.38) |
| Childhood self-control | 0.28  (0.21; 0.35) | 0.27  (0.20; 0.33) |

*Note:* The Table reports associations (expressed as standardized regression coefficients, β with 95% confidence intervals) between parents’ childhood experienced parenting (sensitive parenting and cognitively stimulating environments), cognition and self-control, with parents’ sensitive parenting and cognitively-stimulating environments provided to their own children.
